# Safety and environmental impact control of cross passage construction in soft soil strata using tunnel boring machine method

**DOI:** 10.1038/s41598-023-42082-5

**Published:** 2023-09-14

**Authors:** Ma Yunkang, Yan Wei, Song Yanjie, Zhang Meiqin, Zhou Sheng, Xiong Tianfang, Geng Jia, Li Xiaofan, Cheng Xuesong

**Affiliations:** 1 Tianjin Metro Group Co., Ltd, Tianjin, 300392 China; 2 China Railway, Sixth Survey and Design Institute Group Co. Ltd, Tianjin, 300308 China; 3 School of Civil Engineering, Tianjin University, Tianjin, 300354 China; 4 Key Laboratory of Coastal Civil Engineering Structures and Safety of Ministry of Education, Tianjin, 300354 China

**Keywords:** Engineering, Mathematics and computing

## Abstract

The construction of cross passages using the tunnel boring machine (TBM) method represents an emerging construction technique with numerous advantages. However, owing to the scarcity of application instances, the safety control methodologies and the regulatory patterns concerning environmental impacts remain inconclusive. In this study, a cross passage excavated using the TBM method—the first of its kind in the Tianjin area—was investigated. We identified the key risk control measures for the construction and analysed the TBM operating parameters, monitored ground and building settlements, and monitored mainline tunnel deformations and mechanical responses, revealing the ground and tunnel structure deformation patterns. The following conclusions are drawn. (1) The ground surrounding the cross-passage break-out opening was stabilised by performing secondary grouting and small-range freezing, and the break-in opening was excavated using a completely enclosed steel sleeve. These measures prevented water and sand inflows during the excavation of the break-out and break-in openings in the silt and silty sand strata. (2) The torsional moment of the cutter disc was large during the break-out phase. Break-out mainline tunnel displacement monitoring data indicated that the thrust had a significant effect on the mainline tunnel during the break-out phase. (3) The TBM tunnelling caused ground loss. The ground settlement exhibited a U-shaped distribution along the cross-passage axis, with the maximum settlement being 10 mm. (4) During the break-out phase, the deformation of the break-out mainline tunnel exhibited a duck-egg-shaped distribution. The clearance convergence of the break-out mainline tunnel was within ± 4, and the clearance convergence of the break-in mainline tunnel was controlled within ± 1 mm.

## Introduction

Recently, urban rail transit systems, which are generally constructed underground to minimise disturbance to existing roads and buildings, have been developed rapidly. The twin running tunnels of an underground rail transit line are usually connected with cross passages, which facilitate passenger evacuation, tunnel drainage, and fire prevention and rescue in emergencies, which are typically difficult to deal with in the narrow and almost closed (with few exits) tunnel space^1^. According to the relevant regulations^2^, twin single-track running tunnels should be connected with cross passages that are spaced < 600 m and equipped with parallel Class A fire doors that open in opposite directions and do not intrude into the clearances of the running tunnels when opened.

Traditionally, cross passages have been excavated manually using the mining method after stabilising the soil to be excavated and the surrounding soil using the ground freezing technique. This tunnelling method is relatively mature and has been widely used. However, it has major disadvantages, such as high soil stabilisation costs, vulnerability to thaw collapse, and difficult ground freezing for long cross passages. Additionally, ground freezing for the construction of a cross passage in water-bearing soft soil strata entails a relatively high risk. For example, inadequate ground freezing can easily lead to water and sand leakage, which may have catastrophic consequences. Several large-scale tunnel collapse accidents caused by inadequate ground freezing-induced water and sand leakage have been reported. For example, such accidents have occurred at Shanghai Metro Line 4 and Kaohsiung Metro^3^.

Therefore, to better develop the underground space and minimise the effects of cross passage construction on the environment and existing structures, continuous theoretical and practical efforts have been made towards the advancement of cross passage construction technology. New fully mechanised tunnelling methods that do not require large-scale ground freezing and thus do not have the disadvantages of tunnelling methods requiring large-scale ground freezing, including pipe jacking and tunnel boring machine (TBM) methods, are undergoing rapid development. Currently, the pipe jacking method is the most widely used mechanised method for cross passage construction. It was used to construct the safety passages of the fourth Elbe River Tunnel (Hamburg, Germany), the cross passages of the Emisor Oriente Tunnel (Mexico), the transverse passages of the Tuen Mun-Chek Lap Kok Road Tunnel, and dozens of cross passages in mainland China. The TBM method was first used in China in 2018 to construct the cross passages of the Yinnan section of Ningbo Metro Line 3. Since then, it has attracted increasing attention in China as a new mechanised method for cross passage construction^4^.

Studies have been performed on cross passage construction using mechanised methods. For the key construction techniques of the TBM method, Zhu et al.^5^ summarised the philosophy of cross passage construction using mechanised methods as ‘small-range stabilisation, machinability, strict sealing, and strong support.’ Ding summarised the key techniques of cross passage construction using the TBM method as ‘weak stabilisation, strong support, machinability, total enclosure, constant equilibrium, strict watertightness, and intensive operation.’ Regarding model tests, Zhu et al.^7,8^ conducted full-scale model tests of the T-junctions of cross passages constructed using the TBM and pipe jacking methods and obtained the mechanical responses of the mainline tunnels. Liu et al.^9^ conducted TBM cutting tests using steel-reinforced concrete and composite temporary segments separately. Regarding measurement data analysis, Hu et al.^10^ investigated the effects of the mechanised excavation of a cross passage on the T-junctions through field measurements, theoretical analysis, and finite-element simulations. Mei et al.^11^ investigated the ground settlement associated with a cross passage constructed using a mechanised method using measurement data and found that the ground settlement distribution could be fit with Peck’s curves. Deng et al.^12^ monitored the flexural moments, axial forces, and earth pressures of the lining segments of a cross passage and the break-in mainline tunnel during different phases of the construction process. Wei et al.^13^ carried out field monitoring and discussed the variation rule of ground settlement under the influence of construction combined with theoretical calculation. Regarding case studies of construction projects, Zhu et al.^14^ investigated the external loads and responses of the mainline tunnels of the Ningbo Rail Transit Line 3 during the TBM tunnelling of T-junction cross passages. Ding et al.^15^ analysed the settlement, structural convergence, and structural strains of the mainline tunnels of Wuxi Metro Line 3 during the construction of the cross passages using the pipe jacking method. In addition, researchers ^16–22^ have performed numerical simulations of cross passage construction processes as well as relevant construction techniques and devices using the finite-element method.

The relevant literature indicates that the investigation of cross passages constructed using the TBM method and the analysis of the effects of cross passage construction using this novel method remain inadequate. Additionally, most existing cross passages constructed using the pipe jacking and TBM methods are located in clay strata, which entails a relatively low risk of water and sand leakage during the break-out and break-in phases. The Tianjin area is covered by soft, water-bearing silt and silty sand strata and has a high groundwater table. These complex hydrogeological conditions entail a high risk of water and sand leakage during TBM tunnelling of cross passages—particularly during the break-out and break-in phases. There is a lack of experience and research on the safety control techniques for cross passage construction in such soft soil strata using the TBM method. Therefore, in this study, we investigated the construction of a long cross passage connecting the running tunnels of Tianjin Metro Line 10 using the TBM method, which was located in water-bearing soft soil strata. We systematically summarised the safety control techniques for the TBM tunnelling of the cross passages. We analysed the TBM operating parameters at key points of the construction process, monitoring data of the settlement of the ground and surrounding buildings, and monitoring data of the structural deformation and mechanical responses of the mainline tunnels. Through this, we were able to reveal the ground and structural deformation patterns during the construction of the cross passage, with the goal of providing a reference for popularising the application of this new, advanced tunnelling method.

## Construction project

### Project overview

**Figure 1:**
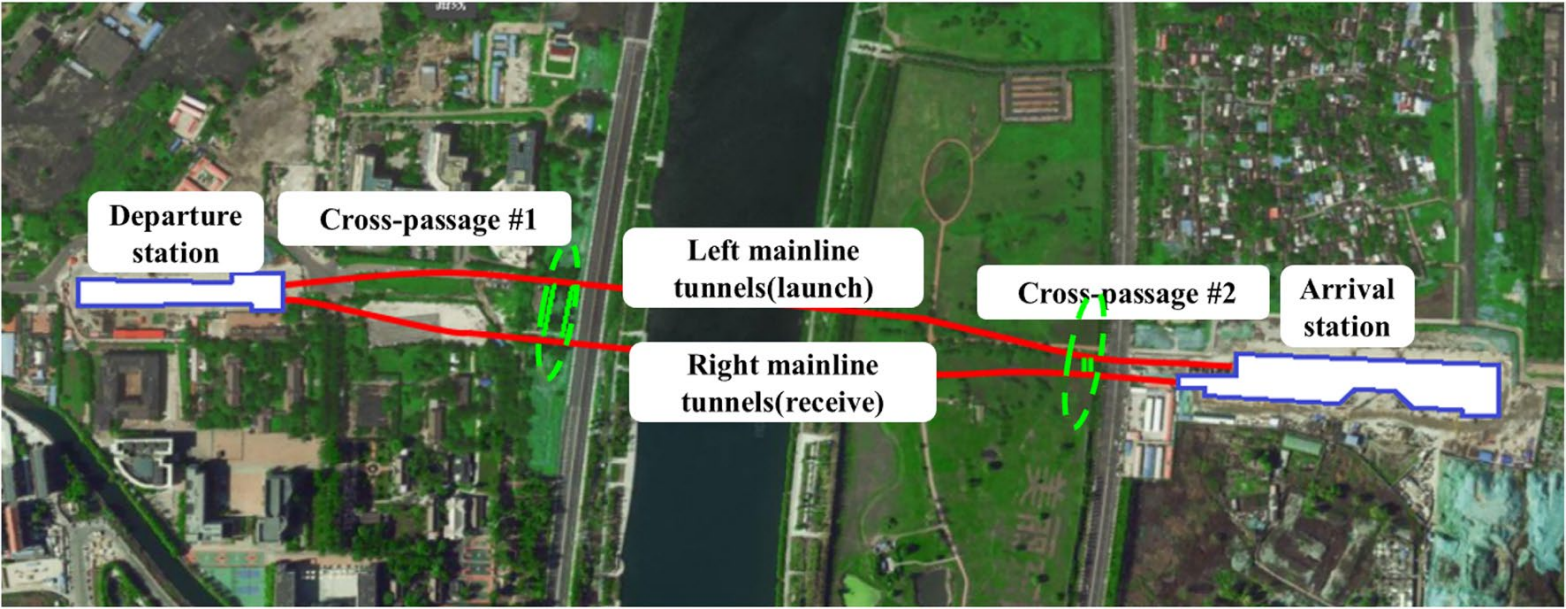
Plan view of the cross passage constructed using the TBM method.

The twin single-track running tunnels of a section of Tianjin Metro Line 10 were investigated, which were constructed using the TBM method at a depth of 11.4–22.4 m. The left and right mainline tunnels were 1095.512 and 1031.102 m long, respectively, and were connected by two cross passages constructed using the TBM method, as shown in Fig. 1. Cross passage #1, which is shown in Fig. 1, was investigated. The central line of the cross passage corresponded to a mileage mark of DK22 + 635.00 on the right mainline tunnel (DK22 + 635.00 on the left mainline tunnel). The angle between the central line of the cross passage and those of the twin mainline tunnels was 90°. The distance between the central lines of the mainline tunnels was 59.0 m. The net length of the cross passage was 52.8 m. Figure 1 shows a plan view of the section of the metro tunnels. The cross passage was covered by an open space (no buildings) directly above. There were a temporarily idled shallow-foundation brick-concrete low residential building near the cross passage and water pipelines (DN1 400) laid (depth = 2.13 m) in the surrounding ground.

**Figure 2:**
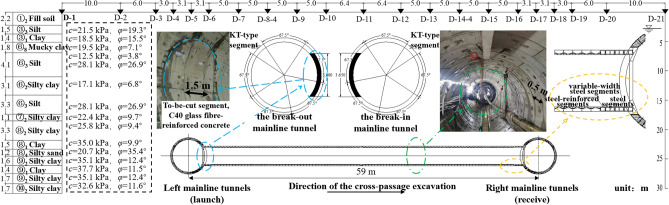
Cross-section and photographs of the cross-passage structure.

The mainline tunnels were constructed using the TBM method. The tunnel lining segments measured 6.2 m in outer diameter, 0.35 m in wall thickness, and 1.5 m in width. The cross passage was constructed using the TBM method. The lining segments measured 3.35 m in outer diameter, 0.25 m in thickness, and 0.5 m in width. A custom-made T3 500 earth pressure balance TBM was used, which was launched from the left mainline tunnel using a semi-closed sleeve and retrieved at the right mainline tunnel using a fully enclosed steel sleeve, as shown in Fig. 2.

For the construction, a soil pressure balanced shield machine with a cutterhead having a spoke-type disc cutter configuration was employed as a TBM. The cutter tools consisted of centre fishtail cutters, ripping cutters, and scraping tools. The TBM excavated with a diameter of 3500 mm, and the cutterhead achieved a maximum rotation speed of 4.7 rpm. The machine exerted a maximum total thrust of 12,000 kN, accompanied by a peak torque of 1009 kN⋅m 2.

The left mainline tunnel was lined using three rings of a ‘steel segment + steel-reinforced concrete segment’ composite lining structure at the break-out of the cross passage, with each ring measuring 1.5 m in width (no wedge). The three rings of special lining segments were jointed with a straight seam, and the joints satisfied pre-set accuracy requirements to ensure an accurate position for the break-out. The break-out opening in the three rings of special segments measured 3.6 m in diameter. The break-in opening measured 3.65 m in diameter. Glass fibre-reinforced concrete with a strength grade of C40 and a waterproofing grade of P10 was used for the to-be-cut part of the composite segmental lining. Steel-reinforced concrete with a strength grade of C50 and a waterproofing grade of P10 was used for the remaining part.

The standard section of the cross passage was lined with steel-reinforced segments. The joints with the mainline tunnels were lined with three rings of steel segments, with each ring measuring 0.5 m in width. The joint with the break-in mainline tunnel was lined with variable-width steel segments to better align them with the joint. The steel-reinforced concrete segments were ordinary wedged segments, with the wedge measuring 9.6 mm. The steel segments were not wedged. All segments were stagger-jointed.

For the construction, a soil pressure balanced shield machine with a cutterhead having a spoke-type disc cutter configuration was employed as a TBM. The cutter tools consisted of centre fishtail cutters, ripping cutters, and scraping tools. The TBM excavated with a diameter of 3500 mm, and the cutterhead achieved a maximum rotation speed of 4.7 rpm. The machine exerted a maximum total thrust of 12,000 kN, accompanied by a peak torque of 1009 kN⋅m.

### Construction process and milestones

The construction of the cross passage started at 9:00 am on 19 April, when the TBM started cutting the break-out segments. On 20 June, the TBM safely entered the retrieval sleeve and stopped advancing. The TBM cutting and excavation process lasted 63 d in total. Table 1 presents the milestones of the construction process.

**Table 1:** Milestones of cross passage construction.

Day	Date (month-date)	Construction task
1	04–19	The TBM was launched in the morning, and it started grinding segments
3	04–21	The cutter disc cut through the mainline tunnel in the evening
9	4–27	The cutter disc completely advanced beyond the segmental lining
21	5–8	Segments were jointed for ring + 1
26	5–13	The shield tail completely advanced beyond break-out opening
50	06–07	The cutter disc reached the segmental ling of the break-in mainline tunnel
54	06–11	The cutter disc started grinding segments
59	06–16	The cutter disc completely advanced beyond the segmental lining
63	06–20	The TBM completely entered the retrieval sleeve

The cross passage was constructed using the TBM method. The construction process was divided into the following phases.

**Figure 3:**
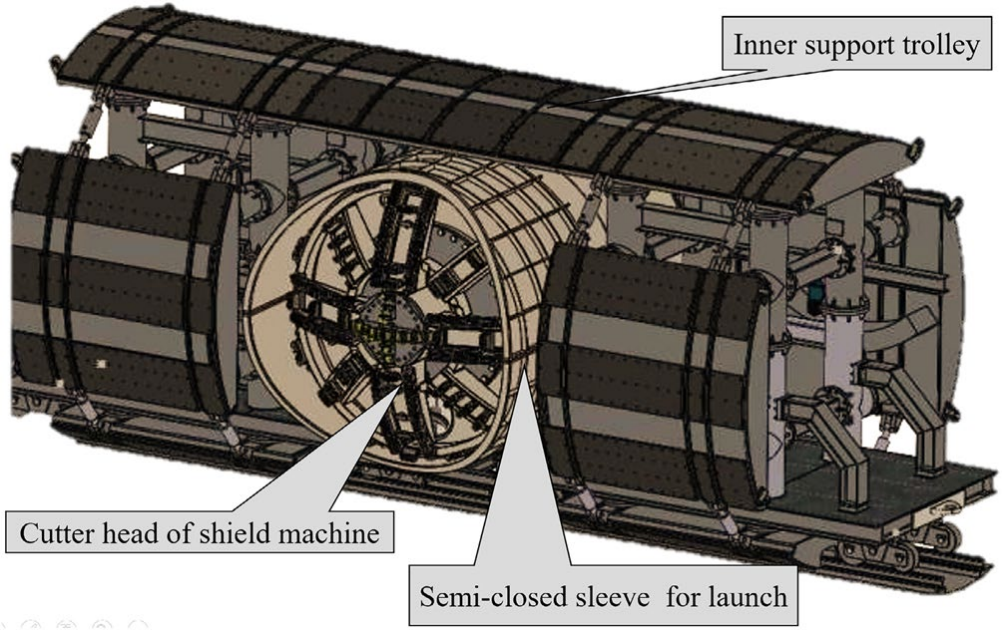
Configuration of mechanical devices for break-out.

**Figure 4:**
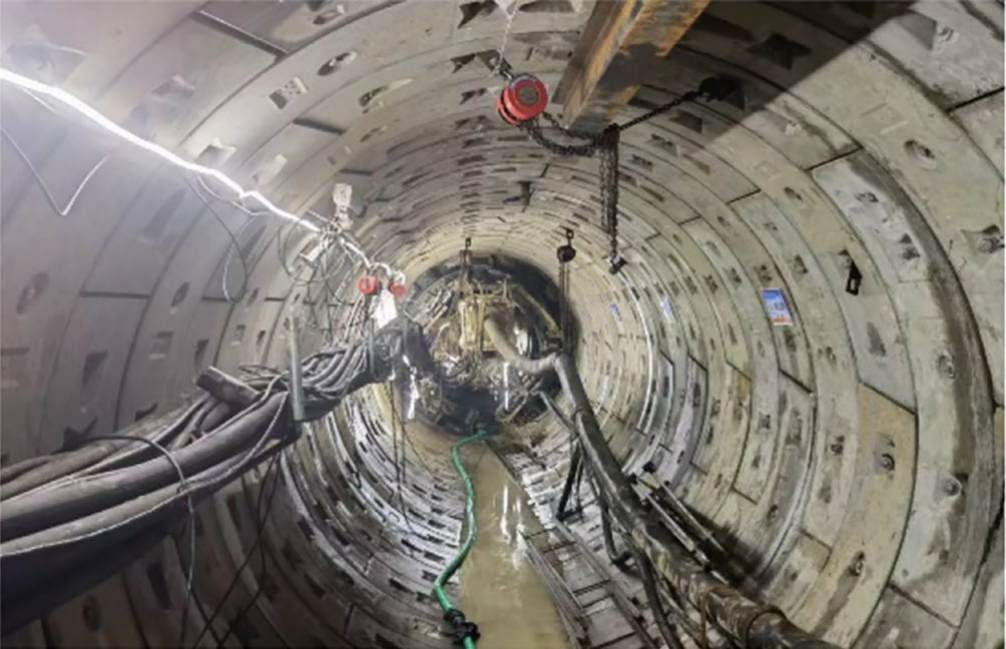
Photograph of normal excavation of the cross passage.

**Figure 5:**
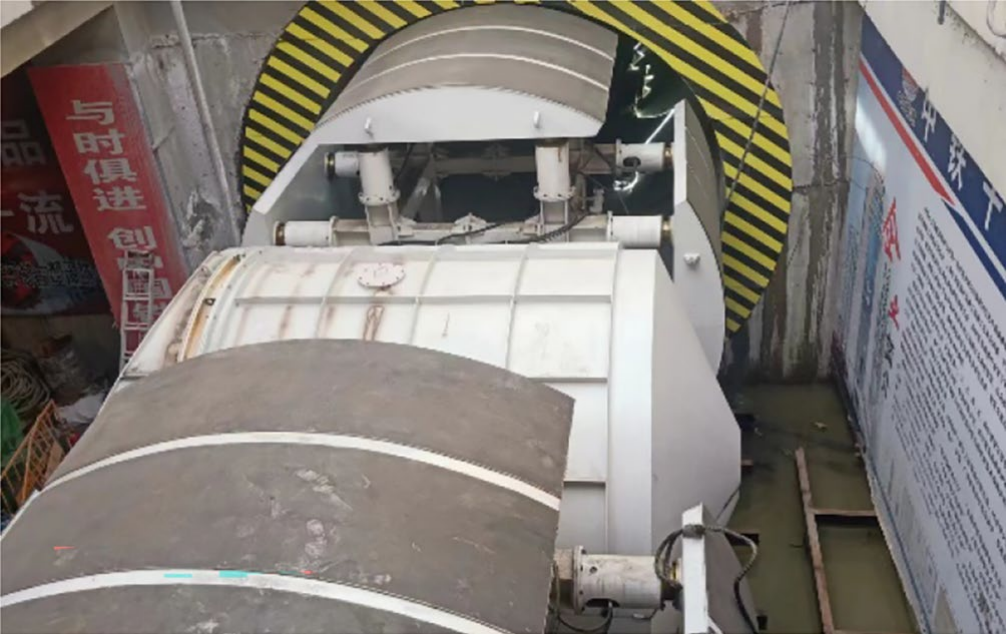
Photograph of the back-up deck and fully enclosed steel sleeve for break-in.

**Figure 6:**
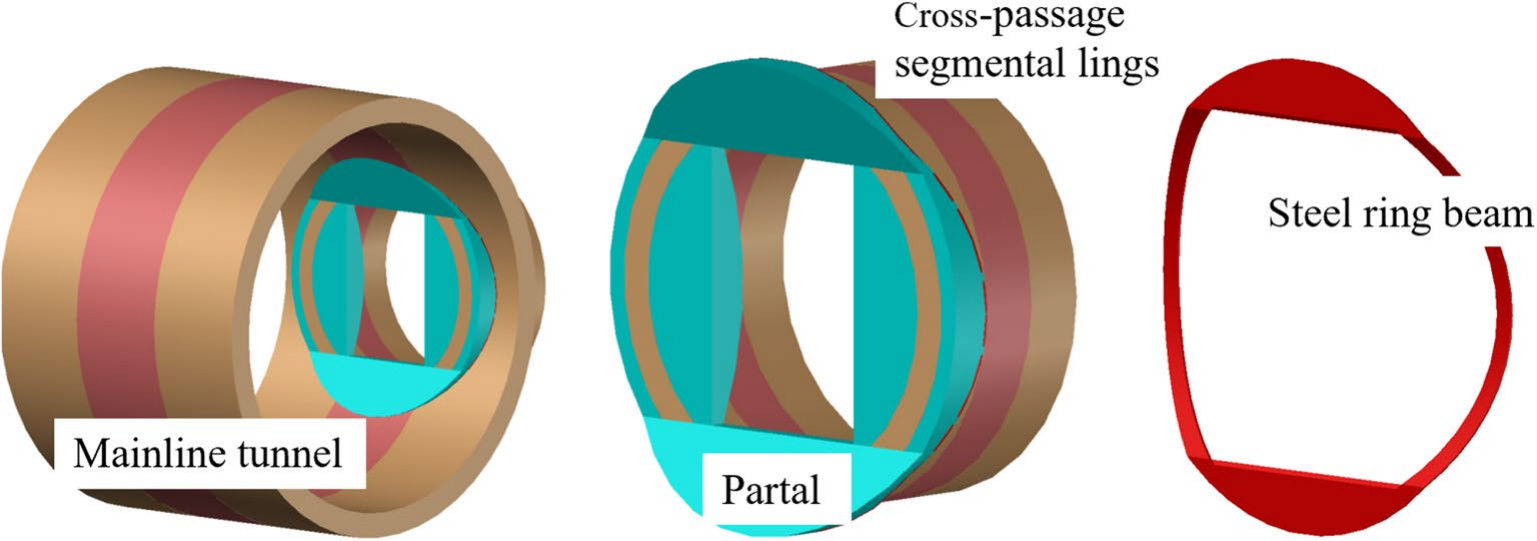
Three-dimensional rendering of the steel-plate ring beam.

(1) Construction preparation phase. The TBM, internal support system, back-up deck, and other devices were transported to position and assembled. A semi-closed sleeve was used for launch. Before launch, the mainline tunnel wall was stabilised via grouting. The sleeve was filled with clay, and bentonite slurry was injected into the sleeve through the cutter head’s muck and water supply system to ensure that the excavation chamber was filled with soil before launch. The position and posture of the internal support system were adjusted to apply a support force. Figure 3 shows a model of the configuration of the mechanical devices during the launch phase.

(2) Grinding break-out segments phase. During this phase, the cutter disc cut the break-out segments. The thrust of the TBM was transferred to the internal support system via a reaction frame. There was a 65-mm void between the TBM and steel sleeve, which was sealed with three steel wire brushes and grease supplied from the shield tail.

(3) Normal excavation phase. After forming the break-out opening, the cutter head of the TBM continued to excavate the cross passage. Secondary grouting performed to stabilise the ground. After the shield tail advanced beyond the break-out opening, the three rings of steel segmental lining of the cross passage at the break-out opening were sealed via grouting. The TBM continued to advance until the cutter disc reached the segmental lining of the break-in mainline tunnel. Figure 4 shows a photograph of the excavation of the cross passage.

(4) Retrieval preparation phase. The receiving deck and sleeve were transported to position. The sleeve was welded and filled with slurry to maintain an equilibrium between the pressure in the chamber and the soil pressure. The entire circumference of the cross-passage break-in opening and the bottom left area of the three rings of the mainline tunnel segmental lining on both sides of the opening was stabilised via grouting to prevent the TBM from tipping over. Figure 5 shows a photograph of the pre-pressurised retrieval sleeve and deck.

(5) Grinding break-in segments phase. The break-in opening was formed using a fully enclosed sleeve. The TBM started grinding the segmental lining. During this phase, the cutter disc thrust, advance rate, and pressure in the sleeve were monitored closely and continuously. The TBM continued to cut the segmental lining until the TBM completely entered the steel sleeve.

(6) Dismantling equipment and constructing openings phase. After the excavation of the cross passage was complete, the soil outside the voids at the openings was sealed via grouting, and the effectiveness of the grouting was inspected. Next, small-range ground freezing was performed using the steel lining segments of the cross passage. Next, the steel sleeve and the temporary segments of the cross passage were removed, and the construction equipment was moved out of the tunnels. Next, the void between the mainline tunnel and cross passage segmental lings was immediately sealed with a rapid-setting sealant, and a steel-plate ring beam was immediately welded at the openings. Finally, the cross passage opening structures were cast in place using a steel bar formwork, completing the construction of the opening structures. Figure 6 shows a three-dimensional rendering of the opening structures.

### Key construction risk control measures

The construction of a cross passage using the TBM method requires adequate technical discussion and construction preparation and involves multiple techniques that must be systematically coordinated to ensure smooth implementation of the construction process ^5^. The excavations of the break-out and break-in openings at the T-junction are two important phases of the construction of a cross passage. During these two phases, the temporary segments are cut away, pending the construction of stabilisation and connection structures at the openings to the mainline tunnels. For a cross passage in water-containing silt or silty sand strata, these two phases are most prone to water and sand inflows. Additionally, when cutting the concrete segmental lining, the TBM was susceptible to tipping over because it was subjected to uneven forces. Therefore, the ground surrounding the openings and the tunnel structure was stabilised and water-sealed using various measures^6^.

**Figure 7:**
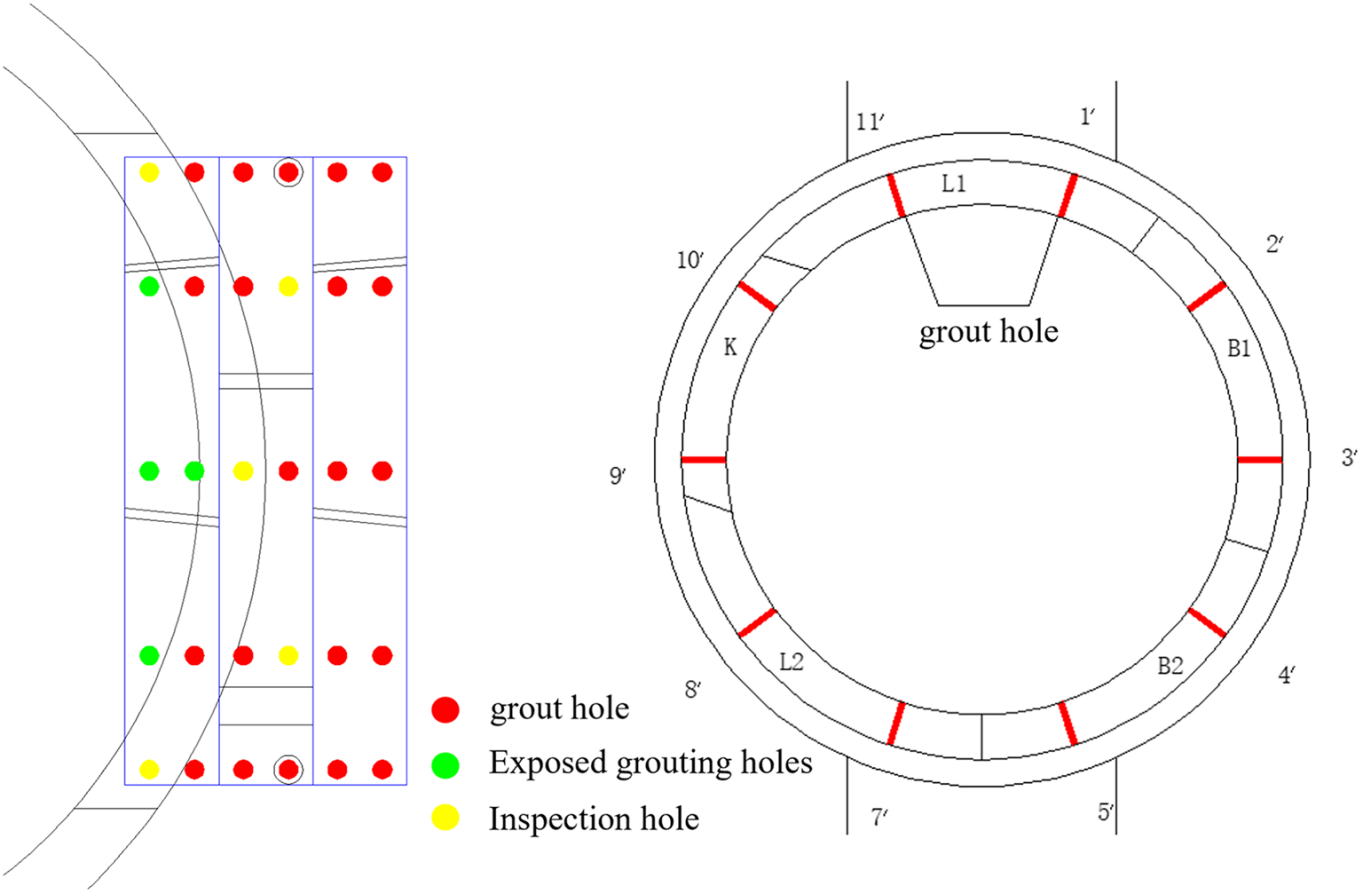
Configuration of grout holes in the steel segments.

(1) To prevent water and sand inflows at the openings, the ground surrounding the openings was sealed via grouting after the formation of the break-out and break-in openings. A total of 20 grout holes were drilled in each ring of the cross passage lining, which were configured in two rings and spaced 36° in the circumferential direction. Figure 7 shows the configuration of the grout holes. The grout holes in the steel segments at the openings exposing the mainline tunnels were not grouted. The grout holes located in the voids at the openings were used as inspection holes.

The first round of grouting at the break-out opening was performed after the shield tail had completely advanced beyond the opening. A total of 9.8 m^3^ of two-liquid grout was injected into the three rings of steel segments at the opening, with the volumes of grout injected through rings + 1, + 2, and + 3 being 1.8, 3.2, and 4.8 m^3^, respectively. The second round of grouting at the opening was performed on 19 June. A total of 19.1 m^3^ of grout was injected, with the volumes of grout injected through rings + 1, + 2, and + 3 being 4.4, 7.7, and 7 m^3^, respectively. After the grouting was complete, its sealing effect was evaluated through the inspection holes. No water seepage or leakage was detected. In addition, the residue on the drill stem and in the inspection holes was cement grout, confirming that the ground at the opening was effectively sealed.

The ground surrounding the break-in opening was grouted after the TBM completely entered the retrieval sleeve. Grout was injected into grout holes drilled in the three rings of steel segments and four rings of ordinary segments at the break-in opening of the cross passage. A total of 14.72 m^3^ of grout was injected (ring + 102: 1.6 m^3^; ring + 103 m^3^: 0.93 m^3^; ring + 104 m^3^: 2.93 m^3^; ring + 105: 1.7 m^3^, ring + 106: 3.6 m^3^; ring + 107: 2.5 m^3^; ring + 108: 1.4 m^3^).

**Figure 8:**
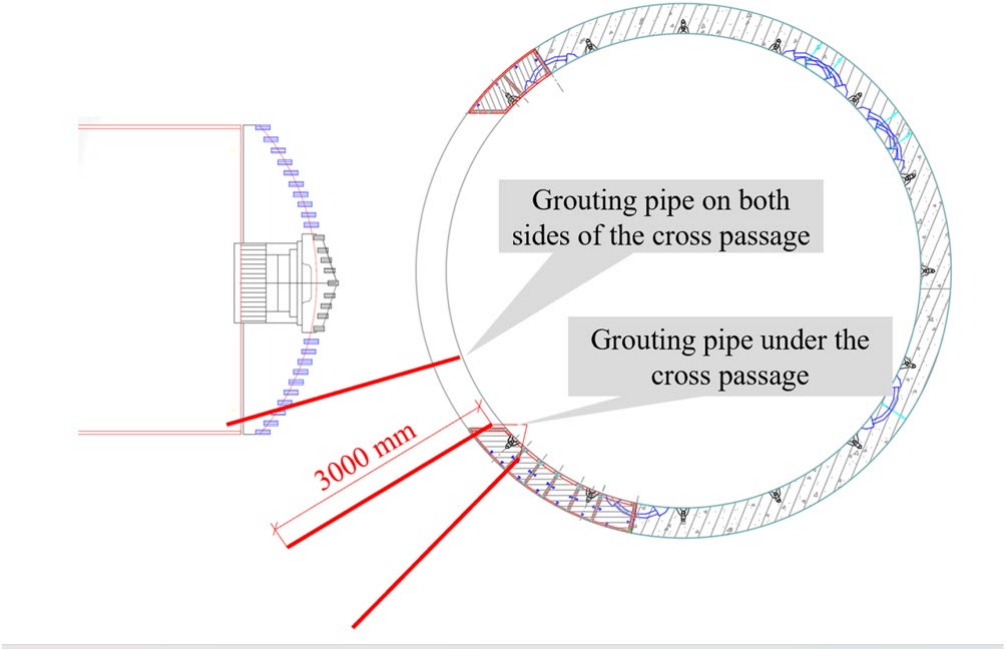
Illustration of the grouted region before excavation of the break-in opening.

**Figure 9:**
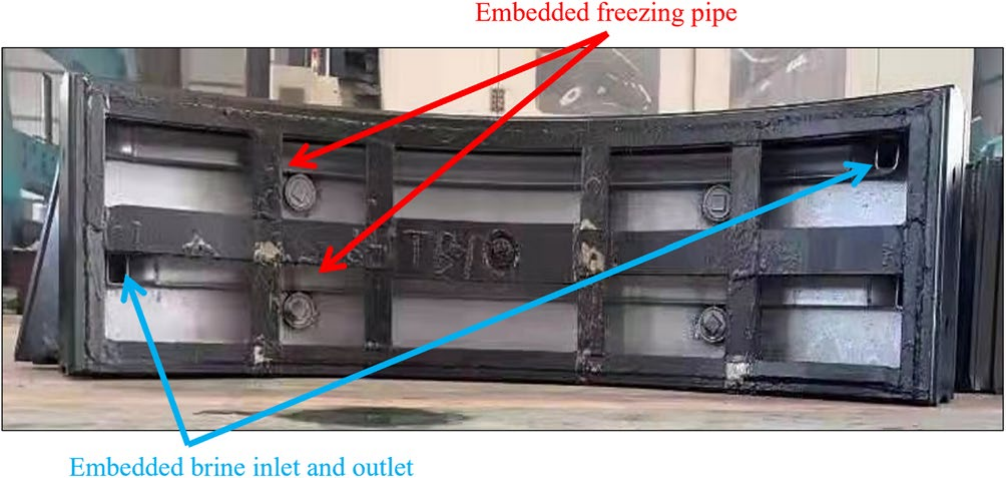
Piping for small-range ground freezing.

**Figure 10:**
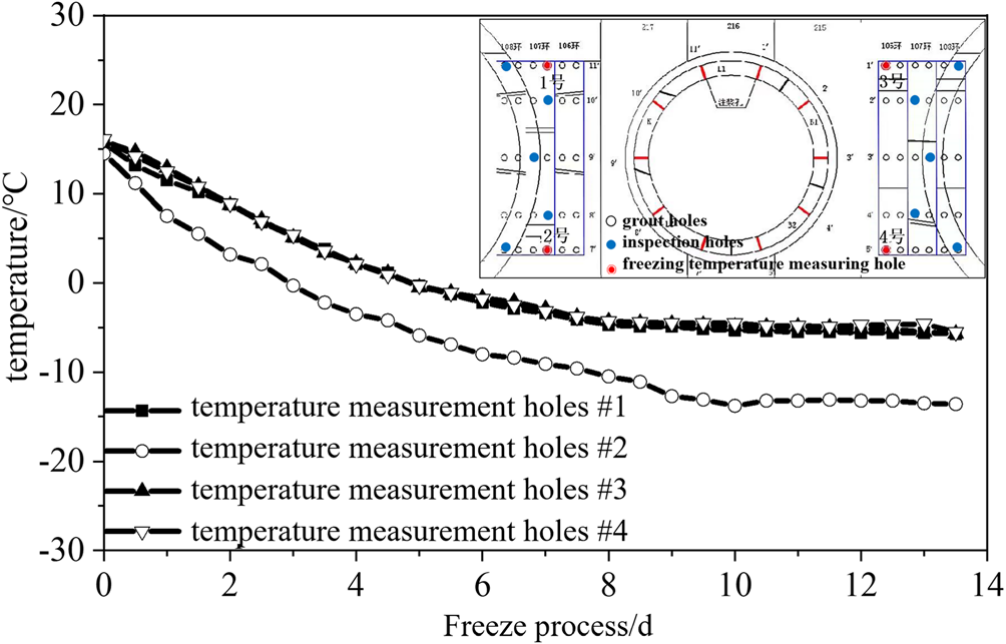
Configuration of the temperature measurement holes and the temperature measurements.

**Figure 11:**
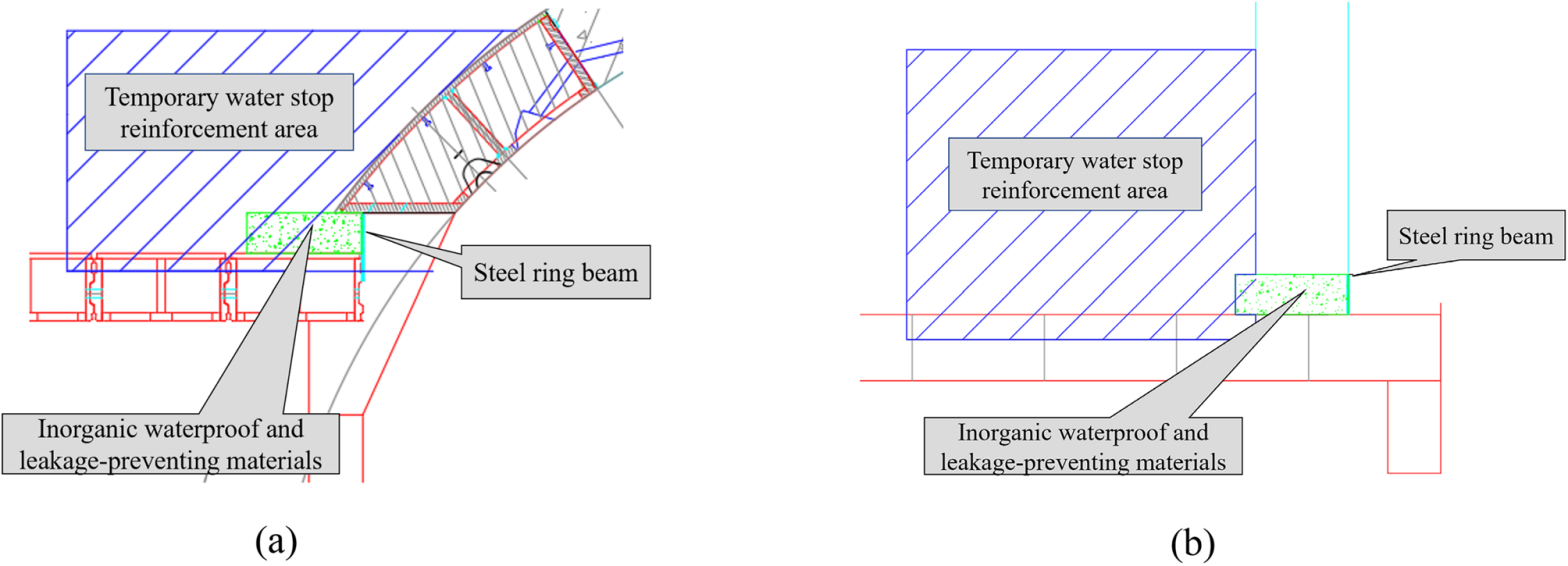
Illustration of the stabilisation of the cross passage–mainline tunnel junction. (**a**) Top of opening, (**b**) Left and right sides of opening.

**Figure 12:**
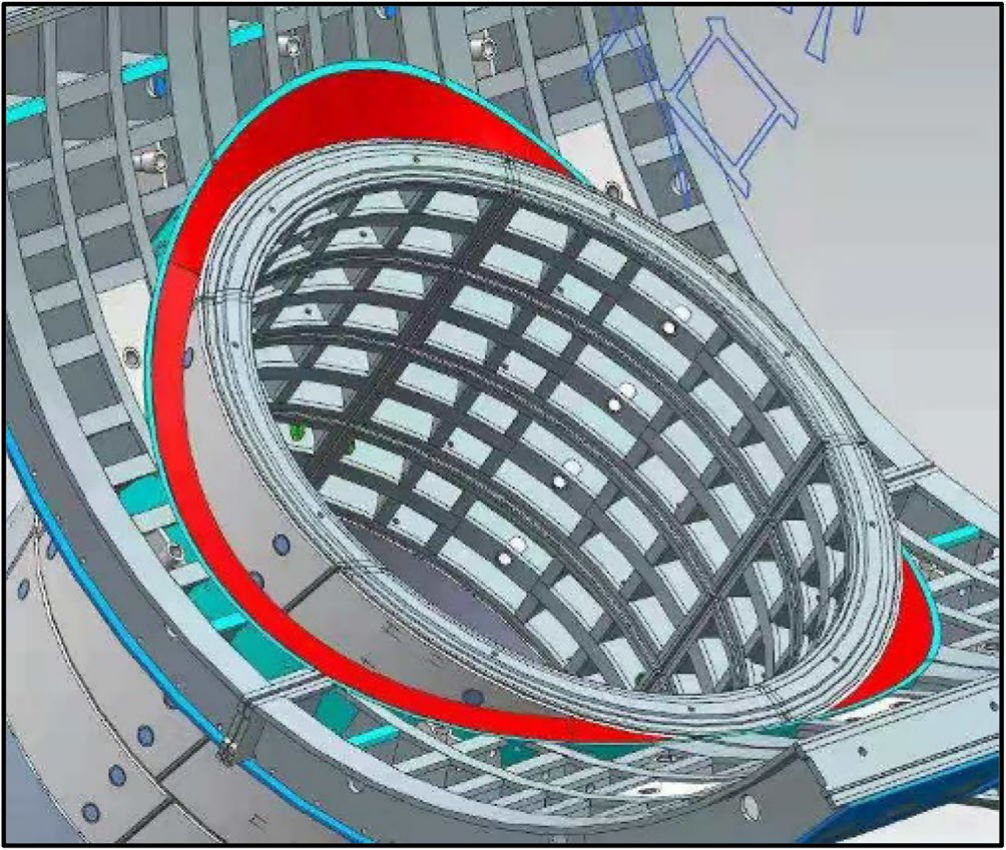
Illustration of the steel-plate ring beam at the opening.

(2) The ground surrounding the break-in opening was grouted before the retrieval sleeve was installed to prevent the TBM from tipping over. Grout was injected into the ground outside the segments via perforated pipes inserted into the lift holes and grout holes pre-drilled in the segments. This stabilised and improved the bearing capacity and the impermeability of the break-in ground. In addition, the posture of the TBM was precisely controlled according to the geometry of the cross passage to prevent the TBM from tipping over during the excavation of the break-in opening. The grouting covered the bottom left region of the three rings of the mainline tunnel segmental lining on each side of the opening, as shown in Fig. 8.

(3) The ground surrounding the junction was stabilised via freezing before the steel sleeve was removed, which served as a temporary water cut-off measure. Traditional tunnelling methods require the freezing-stabilisation of a large region of the surrounding ground, whereas the TBM method requires only freezing-stabilisation of a small region of the ground outside the junctions. Hence, the ground freezing for TBM tunnelling can be referred to as small-range freezing-stabilisation. Rectangular (40 × 60 mm^2^) seamless steel tubes were laid on the inside of the three rings of the steel segmental lining at the break-out and break-in openings as freezing piping with an inlet and outlet for salt brine (Fig. 9). Four grout holes drilled in the segmental lining were reserved for temperature measurement. The temperature was measured up to a depth of 500 mm, as shown in Fig. 10. The soil up to a depth of 500 mm was frozen, satisfying the pre-set requirements.

(4) A steel-plate ring beam was installed at the cross passage–mainline tunnel junctions as a permanent sealing measure after removal of the steel sleeve and temporary segments. As shown in Fig. 11, the arc-shaped sealing steel plate ring beam mainly comprised four parts, which were welded together with watertight seams. The top and bottom plates were welded to the segmental linings of the cross passage and the mainline tunnel at the opening. The side plates were welded to the inner surface of the steel segments of the mainline tunnel and the outer surface of the steel segments of the cross passage. Thus, the mainline tunnel and cross passage were structurally integrated, and the structural integrity of the openings was improved. The opening structure was cast after the construction of the steel-plate ring beam was complete. Figures 11 and 12 show illustrations of the stabilisation and water cut-off measure for the junction and the ring beam, respectively.

## TBM operating parameters

### TBM operating parameters for break-out and break-in phases

According to the relevant literature and construction experience, the TBM was located in the left mainline tunnel during the excavation of the break-out opening. The mainline tunnel served as the carrier of the structure construction force and bore a considerable thrust, which entailed a high risk of excess deformation of the ground surface and mainline tunnel. The cross passage was located in a ⑧_3_ silt stratum with low-pressure artesian water, which entailed a risk of water and sand inflow during the formation of the break-out opening. Therefore, in addition to the necessary soil stabilisation measures described above, the TBM operating parameters were strictly controlled and adjusted during excavation to control the impact of the excavation on the surrounding environment within a safe and reliable range, as shown in Table 2.

**Table 2:** Settings of TBM operating parameters.

Description	Requirements
Posture control	The axis and correction value of the TBM were strictly controlled to prevent it from tipping over
Cutting rate	The cutting rate was set to 4.8 m^3^ (calculated using a lining segment width of 0.5 m and an excavation diameter of 3.5 m). The excavation rate for each ring was controlled at approximately 98% to minimise the disturbance to and maintain the density of the ground
Advance rate	The advance rate was controlled below 5 mm/min for cutting the segmental lining and below 20–25 mm/min during normal tunnelling. The advance rate was adjusted by considering the ground settlement, muck intake and displacement volumes, muck pressure adjusted to maintain an equilibrium with the soil pressure of the working face, and synchronous grouting to minimise over- or under-excavation
Grouting pressure and volume	The grouting pressure was controlled at approximately 0.2–0.3 MPa. The theoretical grouting volume was 0.4 m^3^. The actual grouting volume was set to 200%–250% of the theoretical value and controlled according to the TBM advance rate

The operating parameters were set to values calculated using theoretical equations and were appropriately adjusted according to the actual operating conditions to maintain a dynamical equilibrium between the pressures of the excavation chamber and working face and the stability of the working face. See Table 2 for the relevant control measures adopted during the TBM tunnelling to ensure construction safety.

**Figure 13:**
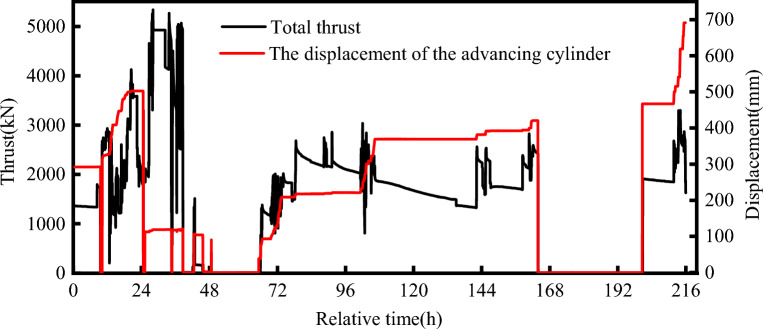
Thrust reaction curve of the TBM during the break-out phase.

The thrust of the TBM was a major factor affecting the deformation and mechanical response of the tunnels. Figure 13 shows the thrust of the TBM in the break-out phase. The thrust curve started at 0:00 on 19 April. The TBM started excavation at 9:00 am, and the thrust increased from 1500 to 5000 kN, which was the maximum during the break-out phase. Then, the reaction frame blocked the tail of the TBM, and the TBM stopped cutting. After the TBM resumed cutting, the thrust was approximately 2000 kN, which was smaller than the previous level. During the break-out phase, the TBM was shut down for repair and maintenance several times because the muck outlet was blocked, resulting in significant thrust variations. The cutting of the segmental lining took a long time, spanning 9 d. The TBM advanced 60 cm during this period. Most of the time was used for repairing the TBM. Table 3 presents the operation record of the TBM. During the break-out phase, the rotation speed of the cutter disc was < 1 rpm, the torsional moment was maintained below 300 kN⋅m, and the advance rate was < 5 mm/min.

**Table 3:** Operation record of the TBM for the break-out phase.

Day	TBM operation record
1	The TBM cutter disc started cutting the mainline tunnel segmental lining and advanced approximately 20 cm on this day
2	The reaction frame blocked the shield tail, and the angle of the reaction frame was adjusted. The TBM advanced 39 mm on this day and 23.9 cm in total
3	The TBM was shut down for repair in the daytime and resumed advancing at 23:00
4	The TBM advanced 14 cm in the early morning and cut through the segmental lining. The muck outlet was blocked in the morning. The gate of the screw machine was blocked by glass fibres, which were cleared away. The TBM advanced 37.9 cm in total
5	The TBM advanced 15 cm in the morning. Then the muck outlet was blocked. The screw machine was retracted to clear the muck outlet. The TBM advanced 52.9 cm in total
6	Work of clearing the muck discharge pipe continued on this day. The TBM advanced 12 mm in the evening. Then, the muck discharge pipe was blocked again
7	Most of the time was used to clear hard concrete blocks. The TBM advanced 5.2 cm on this day and 58.1 cm in total
8	Work of clearing the muck discharge pipe continued on this day
9	The TBM resumed operation at 8:00 pm and advanced approximately 5 cm on this day. The cutter disc completely advanced beyond the mainline tunnel and completed cutting the segmental lining. The TBM advanced 63 cm in total

**Figure 14:**
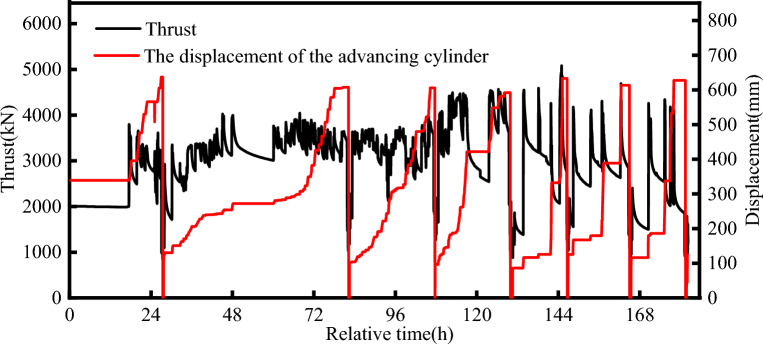
TBM thrust reaction curve for the break-in phase.

Figure 14 shows the thrust of the TBM during the break-in phase. The thrust curve starts at 0:00 on 11 June. At 15 h, the TBM started cutting the segmental lining. The thrust of the TBM increased from 2000 to 3000 kN, and it was maintained at approximately 3500 kN thereafter. At 100 h, the thrust of the TBM began to increase; it reached 4500 kN, and the maximum thrust was observed during break-in phases. To ensure structural stability during segment cutting, the initial advance rate of the TBM was deliberately low. As the cutting of segments progressed, the speed increased. Around the 130-h mark, the cutterhead was fully inserted into the retrieval sleeve, and the TBM commenced cutting the foam concrete within the retrieval sleeve. The thrust gradually decreased to 2000 kN, while the advance rate of the TBM increased to 10 mm/min. The thrust variations were insignificant during this phase. The displacement of the cylinder varied regularly. The TBM advanced smoothly during the break-in phase. The torsional moment of the cutter disc varied between 100 and 200 kN⋅m during the cutting of the segmental lining. The thrust of the TBM during the break-in phase exceeded that in the break-out phase, which is attributed to the different mechanical conditions experienced by the shield machine during the two phases.

### Control of TBM operating parameters during normal excavation phase

**Figure 15:**
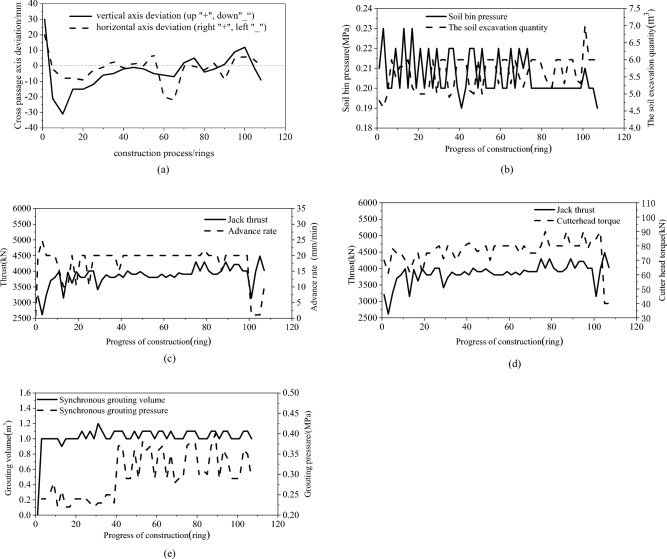
TBM operating parameters for cross-passage excavation. (**a**) Cross-passage axis deviation, (**b**) Excavation chamber pressure and cutting rate, (**c**) Jack thrust and advance rate, (**d**) Cutter disc torsional moment, (**e**) Synchronous grouting pressure and volume.

(1) Fig. 15(a) shows the cross-passage axis deviation curve. During the cross-passage excavation, the horizontal deviation was small, and the maximum horizontal deviation was –22 mm (towards the left), which occurred after the midpoint. The vertical deviation was large after the cutter disc advanced beyond the mainline tunnel during the break-out phase, and the maximum vertical deviation was 30 mm. The vertical deviation was controlled below 10 mm during the later phases. The grouting before the cutter disc entered the mainline tunnel during the break-in phase effectively prevented the TBM from tipping over. Generally, the cross-passage axis deviation was controlled within the specified limit (–100 to + 100 mm).

(2) As indicated by the excavation chamber pressure and cutting rate curves in Fig. 15(b), the excavation chamber pressure varied between 0.19 and 0.23 MPa during the cross-passage excavation. The excavation chamber pressure varied significantly during the early phases, but it varied insignificantly and decreased to the lowest level when the TBM advanced near the break-in mainline tunnel. As the excavation chamber pressure varied, the actual cutting rate varied between 4.8 and 6.0 m^3^, and it was slightly higher than the theoretical cutting rate (4.8 m^3^).

(3) As indicated by the jack thrust and advance rate curves shown in Fig. 15(c) and the cutter disc torsional moment curve shown in Fig. 15(d), the TBM advanced at a smooth rate overall during the cross-passage excavation. During the normal excavation phase, the advance rate varied between 20 and 25 mm/min, the thrust was controlled at approximately 3500 kN, and the cutter disc torsional moment was approximately 80 kN⋅m.

(4) Synchronous grouting was adopted during the excavation to control the soil compressive deformation, increase the soil bearing capacity, and improve the stability of the cross passage segmental lining. As indicated by the synchronous grouting pressure and volume curves shown in Fig. 15(e), the grouting volume was maintained at 1.0 m^3^, which was 250% of the theoretical grouting volume (0.4 m^3^) and within the specified range. The synchronous grouting pressure was 0.2 MPa during the earlier phase of the excavation, and it was increased and varied between 0.3 and 0.4 MPa when the TBM advanced near the midpoint.

## Analysis of monitoring data in construction

### Monitoring program

The cross passage was long and deep underground, with segments located in water-bearing strata. Therefore, the tunnel structure was monitored in real time, and control measures were adjusted accordingly during the excavation to prevent water and sand inflows and other situations that may threaten the environment and the integrity of existing structures.

To understand and provide the variations and operating status of the ground and surrounding buildings and structures and ensure the normal operation of the surrounding buildings, roads, and pipelines, the surrounding environment of the cross passage was monitored, and the monitoring program was optimised according to the monitoring data.

**Figure 16:**
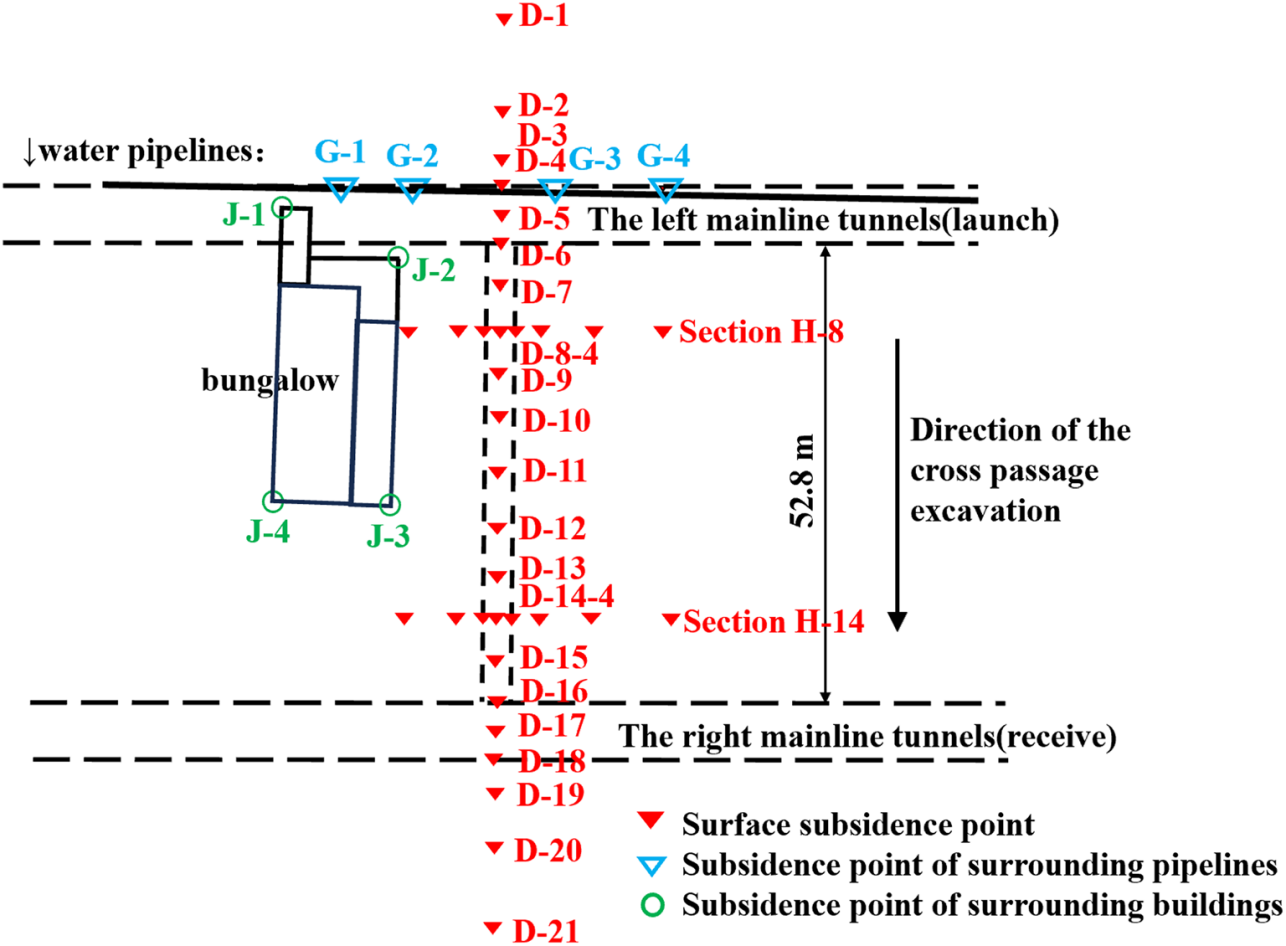
Plan view of the construction site and configuration of monitoring points.

Figure 16 shows the configuration of instrumentation for monitoring the degree and range of the impact of the cross passage construction on the surrounding ground. Monitoring points were set up along the axis and in two transverse cross-sections of the cross passage within 100 m from the break-out and break-in areas and the impact range of risk sources. In addition, the effects of the cross passage construction on the nearby residential building and the water supply pipelines laid in the surrounding ground (material: steel DN1 400; depth: 2.13 m) were monitored.

**Figure 17:**
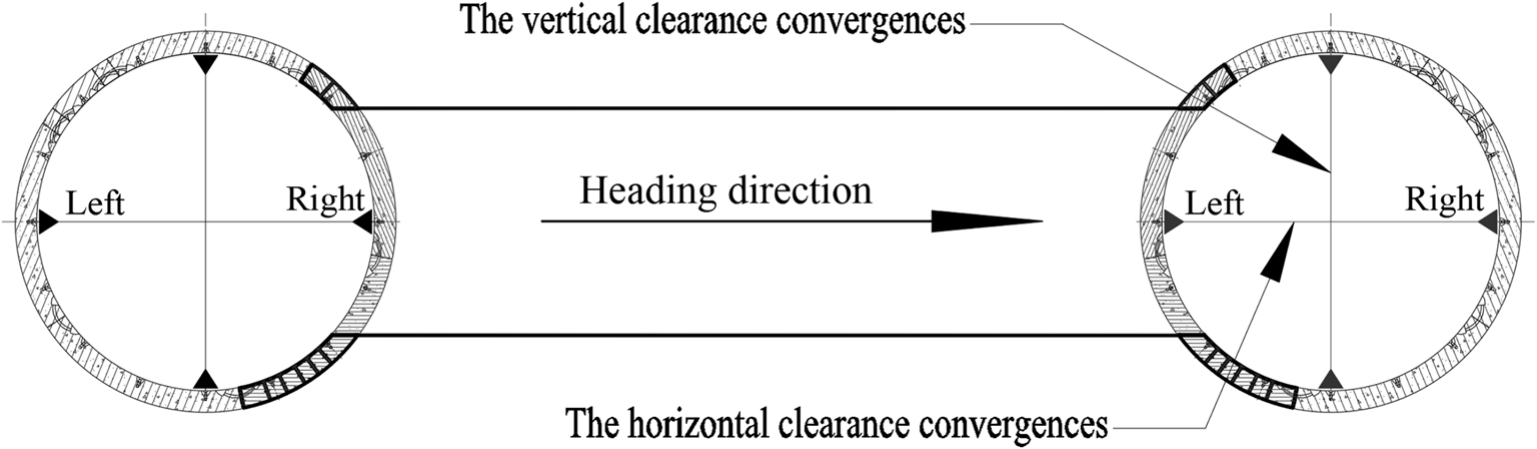
Cross-section of the mainline tunnel.

The horizontal and vertical tunnel clearance convergences and displacements were monitored. The monitoring points were set at the tunnel crown, invert, and sidewalls of each monitoring section. The clearance convergence monitoring points at the crown and invert were also used to monitor the vertical displacement, and those at the tunnel sidewalls were used to monitor the horizontal displacement. Here, GGJ-C and GGJ-S denote the vertical and horizontal clearance convergences of the segmental lining, respectively. GGC denotes the vertical displacement of the invert. GGS-L and GGS-R denote the horizontal displacements of the left and right tunnel walls, respectively. Figure 17 shows the configuration of tunnel structure monitoring points in transverse sections.

**Figure 18:**
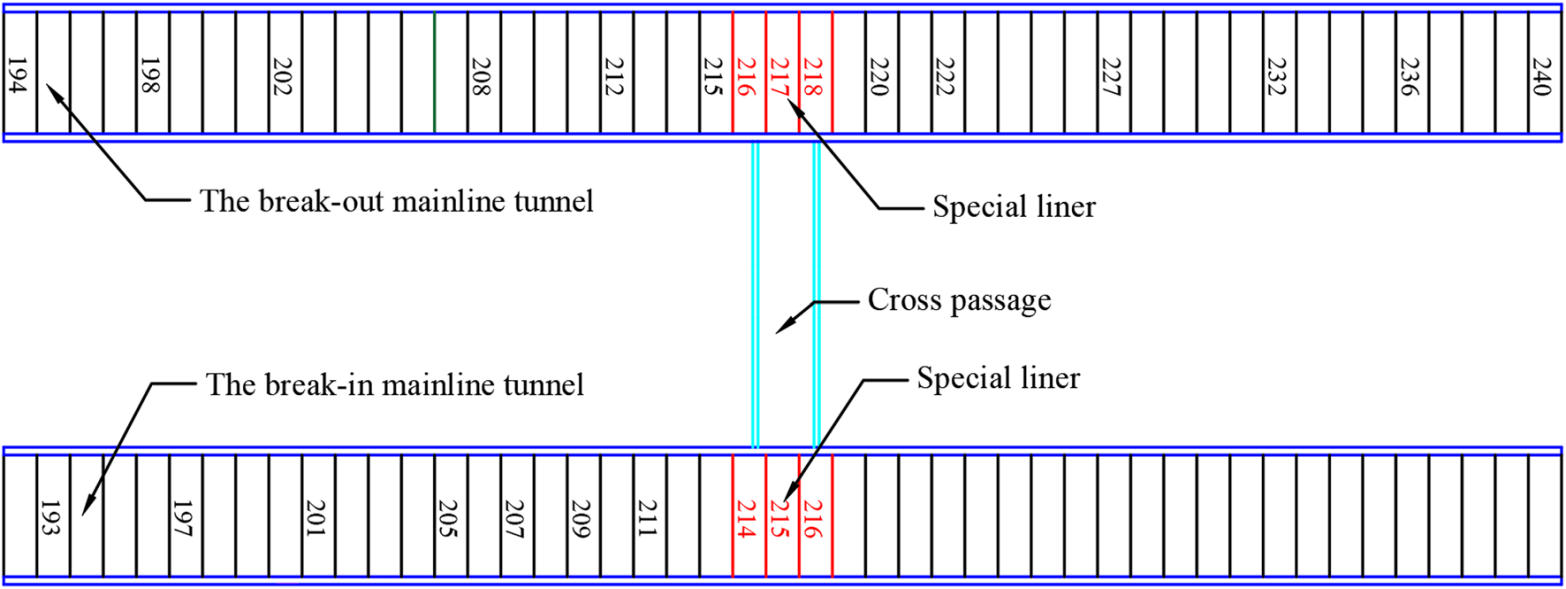
Illustration of segmental lining monitoring points.

The monitoring program could not be implemented at some points during the cross-passage excavation, because access to the monitoring segments was blocked by the back-up deck and other construction devices. Figure 18 shows a plan view of the segmental linings of the mainline tunnels and cross passage. The break-out and break-in openings corresponded to three rings of special segmental lining of the left and right mainline tunnels, respectively (rings 216, 217, and 218 of the break-out mainline tunnel and rings 214, 215, and 216 of the break-in mainline tunnel, with each ring measuring 1.5 m in width).

### Ground and surrounding environment monitoring data

Tunnel excavation causes ground loss. During the cross-passage excavation, the ground settlement at different points was monitored in real time to monitor the degree and range of the impact of the cross-passage excavation on the surrounding environment.

(1) Ground settlement along cross-passage axis.

**Figure 19:**
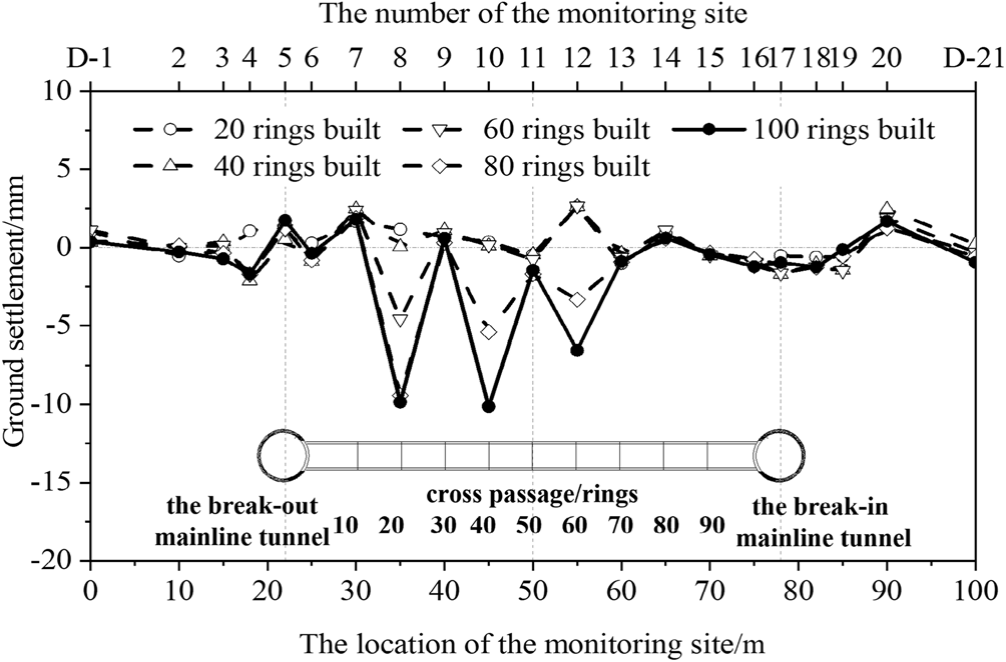
Ground settlement in the cross passage axial cross-section.

The ground settlement along the cross-passage axis was monitored at 21 points (D-1–D-21), as shown in Fig. 19. Different magnitudes of ground settlement were observed in different segments of the cross passage in different phases of the construction process. Significant ground settlement was observed at the middle segment of the cross passage during the normal excavation phase, and the maximum ground settlement was 10 mm. The ground settlements at the break-out and break-in segments were small and stable owing to the internal support system. The ground settlement at some monitoring points varied significantly throughout the construction process. Overall, the ground settlement exhibited a U-shaped distribution along the cross-passage axis.

(2) Ground settlement in cross passage transverse cross-sections.

**Figure 20:**
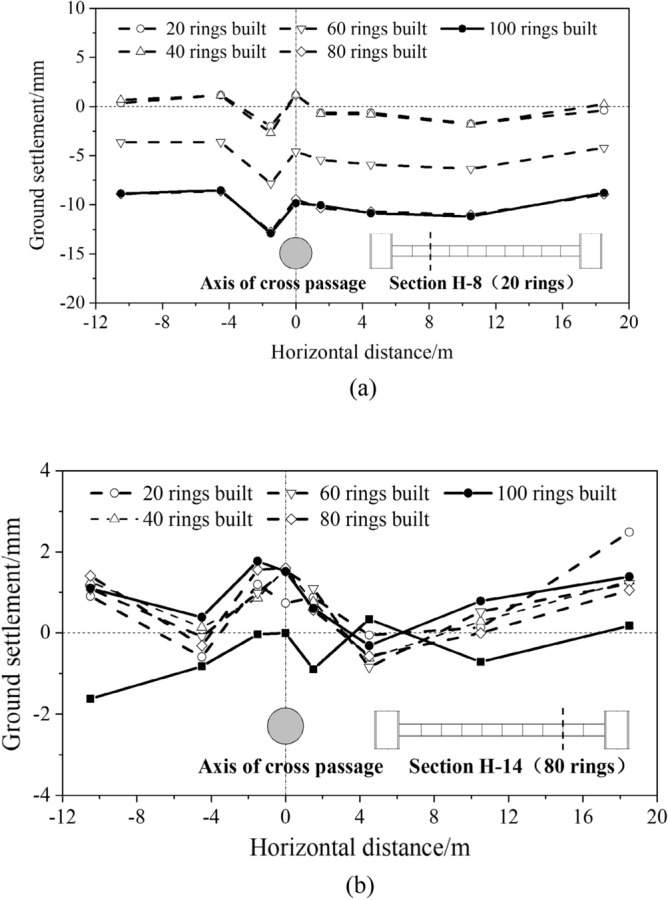
Ground settlement in cross passage transverse cross-sections. (**a**) Monitoring section H-8, (**b**) Monitoring section H-14.

Ground settlement monitoring points were set in monitoring sections H-8 and H-14 (corresponding to rings 20 and 80, respectively). As shown in Fig. 20, a ground settlement of 2 mm was observed directly above the cross passage in monitoring section H-8, but less impact was observed at points farther from the cross passage. The ground settlement exhibited a roughly symmetric distribution in this monitoring section. After the TBM advanced a certain distance from this monitoring section, the impact of the excavation on the ground settlement in this monitoring section decreased. The ground settlement finally stabilised at 10 mm after the grout gelled and dehydrated. For the excavation of a cross passage with an outer diameter of 3.35 m and a depth of 22 m using the TBM method, a ground settlement of 10 mm directly above it indicates a non-negligible impact of the excavation of the cross passage on the ground directly above it. The ground settlement in monitoring section H-14 (located near ring 80 of the cross passage) was small (approximately 2 mm) and stable.

In summary, the ground settlement in the monitoring sections was small during the earlier phase of the cross-passage excavation and increased after the TBM advanced beyond them. The ground settlement exhibited a symmetric distribution along the cross-passage axis. Ground rise was observed above the cross-passage axis owing to grouting-stabilisation.

**Figure 21:**
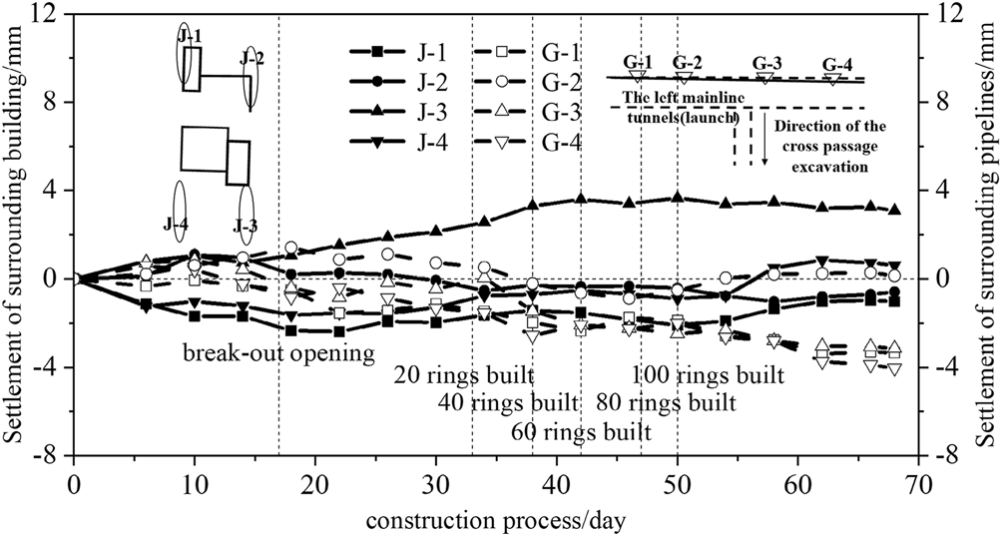
Vertical displacement of pipelines and settlement of building.

The above analysis indicates that the excavation of a cross passage causes ground loss, leading to ground settlement above it and settlement of surrounding buildings—particularly shallow-foundation buildings—and shallow-buried municipal pipelines. Municipal water supply pipelines (material: steel DN1 400; depth: 2.13 m) were laid in the ground near the cross passage (directly above the break-out mainline tunnel). Monitoring points (G-1–G-4) were set up to monitor the deformation of the pipelines. As shown in Fig. 21, during the cross-passage excavation, the maximum cumulative vertical displacement of the pipelines was –4 mm (at G-4), and the average settlement was –2.6 mm.

There were no buildings directly above the cross passage, but there was a low residential building in disrepair near the cross passage. To prevent the cross-passage excavation from causing uneven settlement that could threaten the structural integrity of the residential building, the settlement and differential settlement of the buildings in the length and width directions during different phases of the cross-passage excavation were monitored using monitoring points set at the bottom of the building foundation. Figure 2 shows the location of the building relative to the cross passage. The monitoring points were located in the bearing columns or walls at the four corners of the building.

As shown in Fig. 14, during the break-out phase of the cross-passage excavation, ground rise was observed at monitoring points J-2 and J-3 (located on the side closer to the cross passage), and ground settlement was observed at monitoring points J-1 and J-4 (the side farther from the cross passage). This was possibly due to the thrust of the TBM during the break-out phase, which caused a ground rise in the area closer to the cross passage and a ground settlement in the area farther from the cross passage. When the TBM advanced to a position parallel to the building, the impact of the excavation on the building increased. The ground rise at monitoring point J-3 reached 3.7 mm, but the settlements at other monitoring points decreased and stabilised. Overall, the excavation of the cross passage had a small impact on the shallow-foundation low residential building.

### Monitoring data of break-out mainline tunnel and steel sleeve

**Figure 22:**
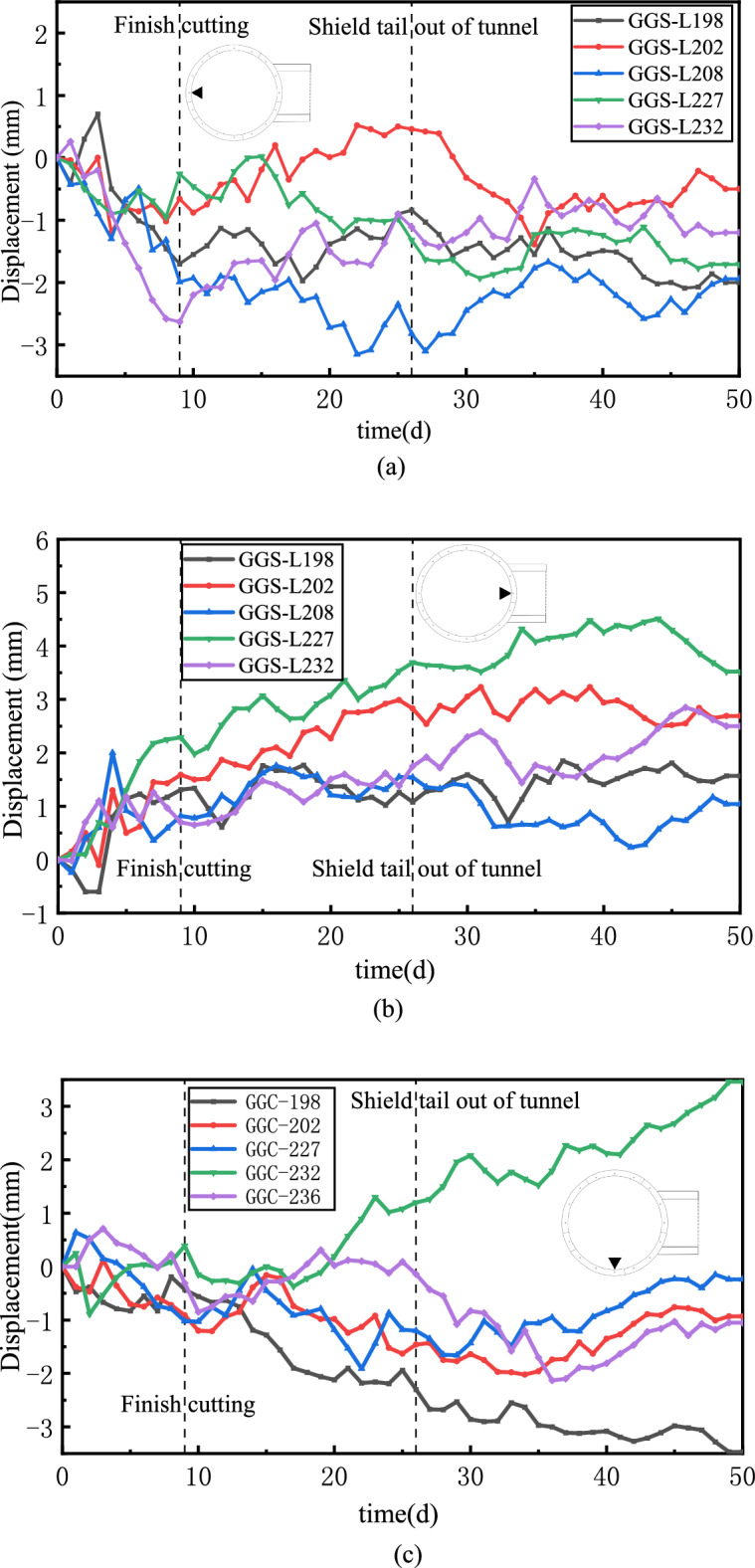
Displacement of the mainline tunnel segmental lining. (**a**) Horizontal displacement of left sidewall, (**b**) Horizontal displacement of right sidewall, (**c**) Vertical displacement of invert.

Figure 22 shows the displacement of the break-out mainline tunnel. The left and right sides of the mainline tunnel corresponded to the rear end of the TBM and the break-out opening of the cross passage, respectively. During the break-out phase of cross-passage excavation, the displacements on the left and right sides increased, and the horizontal displacement of the tunnel exhibited an overall variation trend of the left and right sides moving away from the centre of the mainline tunnel circular cross-section. After the cutter disc advanced beyond the segmental lining and entered the normal excavation phase, the displacement stabilised. The displacement in the break-out phase varied between –3 and 2.5 mm during the break-out phase and between 5 and –4 mm during the entire excavation process. The vertical displacement monitoring data indicated that the tunnel invert subsided, and the maximum settlement was 4.5 mm. The tunnel invert was significantly affected by the back-up deck and other mechanical devices and subsided under the weight of the TBM back-up deck.

**Figure 23:**
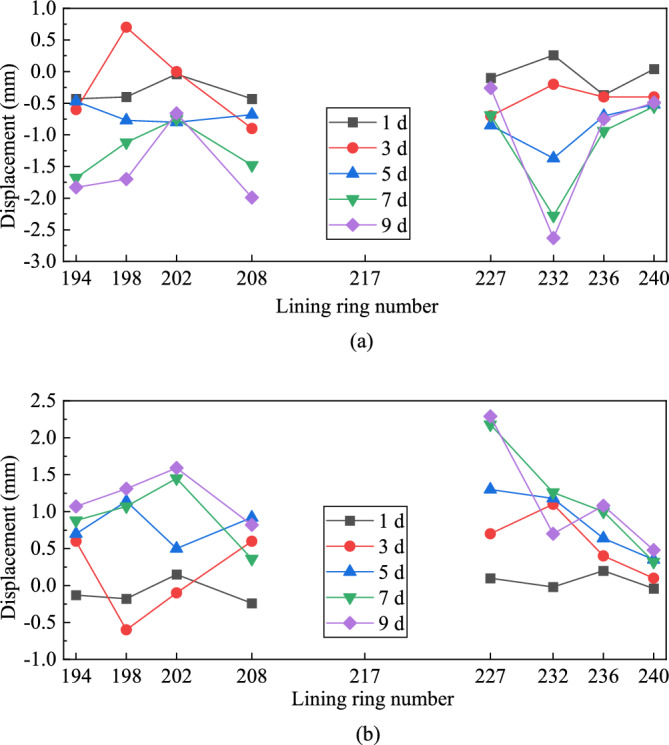
Horizontal displacements of the break-out mainline tunnel sidewalls at different rings. (**a**) Displacement of left sidewall, (**b**) Displacement of right sidewall.

Figure 23 shows the displacements of the sidewalls of the left (break-out) mainline tunnel at different rings. Ring 217 corresponded to the centre of the break-out opening. The displacements of the tunnel sidewalls exhibited an increasing trend from day 1 to day 9. The displacements of the sidewalls from ring 227 to ring 240 exhibited the following variation pattern. The displacement of the segmental lining decreased as the distance to the break-out opening increased. The sidewall displacements from ring 194 to ring 208 fluctuated and exhibited no obvious patterns, possibly owing to the effects of the operation of mechanical construction devices.

**Figure 24:**
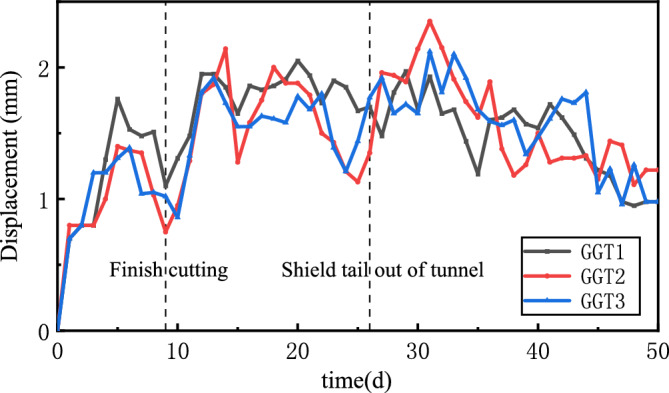
Horizontal displacement of the steel sleeve.

Figure 24 shows the horizontal displacement of the steel sleeve. The horizontal displacement of the steel sleeve was < 2.5 mm throughout the construction period. It increased as the excavation started, stabilised, and decreased after the TBM advanced a certain distance from the break-out opening.

**Figure 25:**
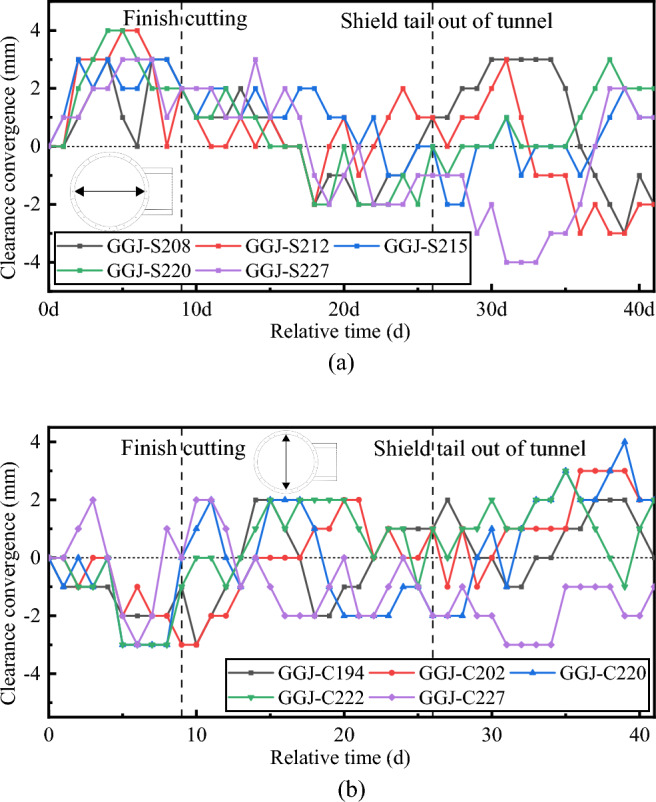
Clearance convergence of the break-out mainline tunnel. (**a**) Horizontal clearance convergence of break-out mainline tunnel, (**b**) Vertical clearance convergence of break-out mainline tunnel.

Figure 25 shows the break-out mainline tunnel clearance convergence curve. During the break-out phase, the horizontal clearance of the mainline tunnel increased at all the monitored rings, with the magnitude of increase being < 4 mm, while the vertical clearance decreased, with the maximum magnitude of decrease being 3 mm. The magnitude of increase/decrease increased when the TBM started cutting the break-out segmental lining and stabilised after the TBM finished cutting the break-out segmental lining. During the break-out phase, the break-out mainline tunnel clearance convergence varied between 4 and –3 mm. Segmental lining ring 194, which was > 40 m from the break-out opening, exhibited outward displacement, indicating that the impact of the cross-passage excavation on the break-out mainline tunnel reached a distance of 50 m from the break-out opening.

### Displacement of break-in mainline tunnel

**Figure 26:**
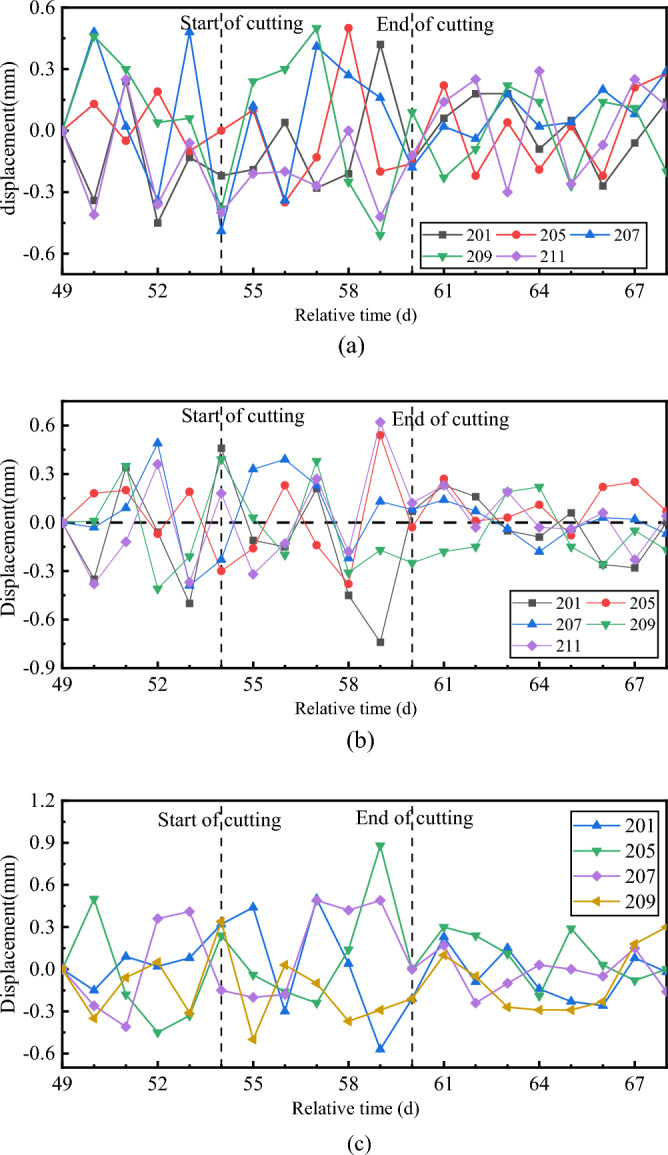
Displacement of the break-in mainline tunnel. (**a**) Horizontal displacement of right sidewall, (**b**) Horizontal displacement of left sidewall, (**c**) Vertical displacement of invert.

Figure 26 shows the monitored displacement of the break-in mainline tunnel. The horizontal and vertical displacements of the tunnel were < 1 mm during the break-in phase. The displacement of the break-in mainline tunnel decreased to < 0.4 mm after day 63, when the TBM cutter disc advanced beyond the segmental lining. This indicated that the TBM’s tunnelling and cutting of the segmental lining affected the break-in mainline tunnel, but the impact was not significant.

The monitored displacements and clearance convergences of the mainline tunnels indicated that the clearance variation and displacement of the break-out mainline tunnel were larger than those of the break-in mainline tunnel. This could be explained by the different structural–mechanical responses of the break-out and break-in mainline tunnels to the cross-passage excavation. (1) When the TBM cut the break-out mainline tunnel segmental lining, the thrust and cutting forces of the cutter disc directly acted on the to-be-cut lining segments of the break-out mainline tunnel, and the reaction of the thrust from the shield tail acted on the back-up deck via the reaction frame and finally was transferred to the break-out mainline tunnel sidewall the via the back-up deck. Thus, the sidewalls of the break-out mainline tunnel were compressed from the inside by two forces in opposite directions. As a result, the mainline tunnel deformed outward in the horizontal direction and inward in the vertical direction. (2) A closed steel sleeve retrieval system was used for the TBM to excavate the break-in opening, and the steel sleeve contained pressurised foamed concrete for maintaining an equilibrium between the pressures on the inside and outside of the opening. (3) During the cutting of the break-in mainline tunnel segmental lining, the transverse horizontal force induced by the soil on the mainline tunnel was gradually replaced by the thrust of the TBM. Consequently, the external force acting on the mainline tunnel did not vary significantly, and the overall displacement of the mainline tunnel was small.

## Conclusion

This paper presents a case study of a long cross passage constructed in soft soil strata using the TBM method. We examined the risk control methods for the construction of cross passages in soft soil strata using the TBM method, key aspects of the construction process, and the impacts on the surrounding environment and tunnel structure, with the goal of providing a reference for similar construction projects. Our conclusions are summarised as follows.

(1) The secondary grouting and small-range freezing implemented at break-out and break-in openings were effective. These measures can be applied in subsequent analogous engineering projects.

(2) The thrust of the shield machine during the normal excavation phase and break-in phase should exceed that during break-out phase. The torque of the cutterhead was higher when it cuts segments during break-out and break-in phases than during the normal excavation phase. Considering the construction outcomes, it is evident that the TBM excavation parameters fall within reasonable ranges.

(3) The excavation of the cross passage using the TBM method caused ground loss, and the ground settlement increased as the excavation work proceeded. The ground settlement along the cross-passage axis exhibited a U-shaped distribution, with the maximum settlement being 10 mm. The ground settlement in cross passage transverse cross-sections increased after the TBM advanced beyond them and exhibited a roughly symmetric distribution around the cross-passage axis.

(4) The excavation of the cross passage caused structural deformation of the mainline tunnels. The deformation of the break-out mainline tunnel exhibited a clear pattern: an overall duck egg-shaped distribution. The clearance convergence of the break-out mainline tunnel was controlled within ± 4 mm. The impact of the TBM cutting the break-out segmental lining on the break-out mainline tunnel structure reached 40 m from the break-out opening.

(5) The displacement and clearance convergence of the break-in mainline tunnel were controlled within ± 1 mm. The impact of the cross-passage excavation on the break-out mainline tunnel was larger than that on the break-in mainline tunnel, for the following reason. During the excavation of the break-out opening, both the thrust of the TBM and the reaction of the thrust acted on the break-out mainline tunnel, and the two horizontal forces in opposite directions acted on the left and right sidewalls of the mainline tunnel, respectively. As a result, the tunnel deformed outward in the horizontal direction. In contrast, during the excavation of the break-in opening (when a totally enclosed steel sleeve was used), the water and soil pressure acting on the outer side of the partial segments were gradually replaced by the thrust of the TBM. Consequently, the forces acting on the mainline tunnel were maintained in a dynamic equilibrium.

## Data Availability

All data generated and analysed during the study are included in this article.
